# Effects of different rehabilitation modality on cardiopulmonary function in patients with acute coronary syndrome after revascularization

**DOI:** 10.3389/fcvm.2023.1120665

**Published:** 2024-03-04

**Authors:** Wanping Chen, Yan Feng, Meili Yu, Zhaoguo Zhang, Jiahui Wu, Wenxian Liu, Wei Gu

**Affiliations:** ^1^ Cardiac Rehabilitation Center, Department of Cardiology, Beijing Anzhen Hospital, Capital Medical University, School of General Practice and Continuing Education, Capital Medical University, Beijing, China; ^2^ Cardiac Rehabilitation Center, Beijing Hospital of Integrated Traditional Chinese and Western Medicine, Beijing, China; ^3^ Coronary Heart Disease Center, Department of Cardiology, Beijing Anzhen Hospital, Capital Medical University, Beijing, China

**Keywords:** acute coronary syndrome, cardiopulmonary exercise test, cardiac rehabilitation, home-based rehabilitation group, center guided home-based rehabilitation group

## Abstract

**Objective:**

To investigate the effects of different rehabilitation modalities on cardiopulmonary function in patients with acute coronary syndrome after revascularization.

**Methods:**

Two randomized controlled trials were conducted. All patients were stable for more than 48 h and less than 1 week after revascularization for acute coronary syndrome and were randomly assigned to Group A (home-based rehabilitation group) or Group B (center guided home-based rehabilitation group). The cardiopulmonary exercise test was mainly performed before and 3 months after cardiac rehabilitation (at the end of intervention). The primary endpoints of the study were peak oxygen uptake (VO2peak), and the secondary endpoints were maximum metabolic equivalents (METs), anaerobic threshold exercise load (Load AT), maximal workload (Load max), and anaerobic threshold oxygen uptake (VO2 AT).

**Results:**

A total of 106 patients were included in the study, with 47 patients in Group A (with 6 losses) and 50 patients in Group B (with 3 losses). There were no significant difference between the two groups in terms of age, gender, body mass index (BMI), left ventricular ejection fraction(LVEF), low-density lipoprotein cholesterol(LDL-C),cardiovascular risk factors. In Group A, no significant differences in CPET indices were observed before and after the intervention. In Group B, values of maximum metabolic equivalents (METs), peak heart rate (PHR), anaerobic threshold exercise load (Load AT), maximal workload (Load max), maximum ventilation per minute (VE max), peak oxygen uptake (VO2peak), anaerobic threshold oxygen uptake (VO2 AT) and maximum oxygen pulse (VO2/HRmax) were higher than those before the intervention (*P* < 0.05). In addition, METs (max), Load AT, Load max, VO2 AT, and VO2peak in Group B were higher than those in group A (*P* < 0.05). The change rates of VO2peak, METs(max), PHR, Load max, VO2 AT, VE max, VO2/HR(max) in the two groups were significantly different before and after intervention (*P* <  0.05).

**Conclusion:**

Cardiac exercise rehabilitation is helpful for improving patients’ cardiopulmonary endurance and quality of life. Moreover, rehabilitation modalities with regular hospital guidance can improve cardiopulmonary function in a shorter period,which seems to be more effective than a complete home-based rehabilitation model.

**Clinical Trial Registration:**

http://www.chictr.org.cn, identifier (ChiCTR2400081034).

## Background

Acute coronary syndrome (ACS) (1) is one of the leading causes of death in patients with coronary heart disease. According to a report on Cardiovascular Health and Diseases in China in 2020, the prevalence of cardiovascular diseases in China is increasing. An estimated 330 million people have cardiovascular diseases, including 11.39 million cases of coronary heart disease. After more than 1 month of complete coronary revascularization, morbidity, mortality, and readmission rates for ACS remain high.

As a widely accepted treatment for the secondary prevention of coronary heart disease, cardiac rehabilitation (2, 3) is a quantifiable and executable noninvasive clinical practice system of cardiology that integrates cardiovascular medicine, sports medicine, rehabilitation medicine, nutrition, psychology, behavioral medicine, and preventive medicine. Many clinical trials and guidelines (3–7) have confirmed the positive impact of cardiac rehabilitation on patients with coronary heart disease. It improves cardiovascular function and exercise capacity, reducing recurrence (8) and rehospitalization rates (9) and ultimately improving the quality of life of patients (10). However, patients' referral rate (11), participation rate, and compliance (12) remain low. The reasons for this remain unclear, but may include an imperfect hospital referral system, limited coverage of medical insurance, and insufficient awareness among patients (13, 14). Currently, telemedicine technology has provided a feasible solution for implementing home-based remote cardiac rehabilitation (15). Several foreign studies (16, 17) have shown that patients undergoing home-based cardiac rehabilitation can achieve the same effects as those receiving cardiac rehabilitation in hospitals. The home-based cardiac rehabilitation mode is not limited by space or time and can be used as an effective alternative to traditional hospital-based cardiac rehabilitation (18, 19) to expand accessibility and participation in cardiac rehabilitation, improve cost-effectiveness (20), and increase the long-term benefits (21) of cardiac rehabilitation for patients with cardiovascular diseases.

The cardiopulmonary exercise test (CPET) (22) is a non-invasive assessment method for cardiopulmonary function and can objectively, comprehensively, and quantitatively assess patients' cardiopulmonary reserve capacity and exercise tolerance. It has important clinical application value in the diagnosis of cardiopulmonary diseases, disease risk stratification, clinical efficacy, prognosis evaluation, and cardiac rehabilitation guidance.

Therefore, we conducted a trial to compare improvements in cardiopulmonary function in patients with ACS after two different models of cardiac rehabilitation, namely home- based rehabilitation and center guided home-based rehabilitation, to better understand how patients benefited from cardiac rehabilitation and provide reference values for adjusting and optimizing the cardiac rehabilitation mode.

## Materials and methods

### Research participants

From April 2021 to September 2021, all patients in stable conditions for more than 48 h and less than 1 week after complete coronary revascularization for ACS were admitted to the cardiac rehabilitation centers of Beijing Anzhen Hospital and Beijing Hospital of Integrated Traditional Chinese and Western Medicine. This trial was approved by the hospital's ethics committee, and all patients signed the informed consent forms (KS2021165). The trial was registered at http://www.chictr.org.cn.

The inclusion criteria were as follows: age between 35 and 70 years, coronary angiography-confirmed ACS, after complete coronary revascularization, determined treatment plan, normal markers of myocardial injury, stable patient condition, and no risk of sudden death caused by malignant arrhythmia. Exclusion criteria were new ischemic symptoms clearly observed on resting electrocardiography, uncontrolled arrhythmia leading to symptoms or hemodynamic disorders, unstable angina pectoris, decompensated heart failure, active endocarditis including subacute myocarditis or pericarditis, acute non-cardiac diseases such as infection, renal failure, and hyperthyroidism, acute pulmonary embolism or pulmonary infarction, quiet heart rate >120 beats/min (including transient increase), incompatibility of patients, electrolyte abnormalities, bradycardia or tachycardia, systolic blood pressure > 200 mmHg and diastolic blood pressure > 100 mmHg at rest, complicated ventricular arrhythmia, severe valvular disease, hypertrophic heart disease or other outflow tract obstruction, serious pulmonary hypertension, patients with degree III atrioventricular block or their family members not agreeing to participate in the study, and patients who withdrew from the research program or were lost to follow-up.

### Interventions

After enrollment, the clinical data of all the patients, including age, sex, body mass index, left ventricular ejection fraction, low-density lipoprotein cholesterol level, hypertension, hyperlipidemia, and diabetes, were recorded. All patients underwent routine secondary preventive treatment, including drug prescription, nutrition prescription, psychological prescription and smoking cessation prescription.

Before cardiac exercise rehabilitation, patients underwent CPET to evaluate their cardiopulmonary reserve capacity and exercise tolerance. All CPETs were performed by a certified professional (clinician, cardiac rehabilitation center personnel, or caregiver). Before the start of the project, the participating technicians shall be trained uniformly to ensure the homogeneity of the operators. All the instruments adopt the German Jaeger Exercise Cardiopulmonary Testing System (AT-104HS-ERGO).

Next, a personal exercise prescription was created, including the exercise method, intensity, time, frequency, and progress. Moreover, Adjustment of prescription shall follow the principle of gradual progress: adjust the exercise program once a week; only one item of exercise prescription (such as time, frequency, intensity, etc.) is adjusted at a time; increase the duration of aerobic exercise for 1–5 min at a time until reaching the target value; increase the strength and duration by 5%–10% each time, which is generally well tolerated; increase the duration of aerobic exercise to the expected goal at first, and then increase the intensity and/or frequency.

The classic exercise rehabilitation program was divided into three steps: preparation, training and relaxation. The warm-up activities was to use low-level aerobic exercise for 5–10 min to relax and stretch muscles, improve joint mobility and cardiovascular adaptability, and prevent adverse cardiac events induced by exercise and sports injuries. The training stage included aerobic exercise, resistance exercise, flexibility exercise and balance exercise. The forms of aerobic exercise included walking, jogging, cycling, swimming, aerobics, climbing stairs, dancing, some ball games, pedaling and rowing on equipment, etc. Resistance exercise could exercise muscle strength and endurance through elastic belt. The time of aerobic exercise could be gradually increased from the initial 20 min to 40–60 min. Resistance exercise, flexibility exercise and balance exercise were 5–10 min each time. The exercise intensity was determined by the target heart rate [target heart rate = (maximum heart rate-resting heart rate) × 40%–60%+resting heart rate] and rating of perceived exertion. The required intensity of motion was subjectively measured using a Borg score of 12–16. For patients with poor physical fitness, the exercise intensity level was set at 40%, and with the improvement of physical fitness, the exercise intensity was gradually increased. For patients with good physical fitness, the exercise intensity could be set to 60%. The frequency of exercise was 3–5 times a week. Relaxation could be a continuation of slow-paced aerobic exercise or flexibility training, which could last for 5–10 min.

After baseline assessment, patients were randomized into Group A or B. Group A focused on home-based cardiac rehabilitation. Video explanations and standardized training actions were performed remotely through the Internet. Health education and weekly one-on-one Q&A sessions were performed via the WeChat Official Account. The patient's personal exercise log was established to record, guide, and supervise the implementation of the exercise rehabilitation plan, and timely feedback and communication were provided to adjust exercise rehabilitation prescriptions. Monitor whether the patient's heart rate during exercise reaches the effective range of the target heart rate required by the individualized prescription through the smart bracelet, and let the patient upload the heart rate record chart of the bracelet during the follow-up for regular quality control. The patients were required to perform aerobic exercise at least Three times a week for a minimum of 30 min each time for home-based cardiac rehabilitation. Group B is hospital-led family rehabilitation. They received exercise rehabilitation training once a week at the hospital under the guidance of a cardiac rehabilitation professional, who provided timely feedback to improve exercise prescriptions. The remaining patients received cardiac rehabilitation at home, similar to Group A.

Both groups received exercise rehabilitation therapy 3–5 times a week for 12 weeks. Subsequently, CPET was repeated to evaluate the curative effects of cardiac rehabilitation exercises on patients in the two modalities.

### Measurements

In the CPET, the symptom-limiting exercise test was performed using a treadmill with a continuous increasing power scheme, followed by cardiopulmonary function measurement at resting state for 3 min and a warm-up exercise without power load for

3 min. Subsequently, the initial treadmill speed was set to 60 rpm. According to the patient's sex, age, basic disease, functional status, and exercise habits, exercise with increasing power cycling load was selected so that the patient could achieve symptom- limiting exercise within 8–12 min and resume exercise in the last 5 min.

### Observation indicators

CPET indicators included maximum metabolic equivalents (METs), peak heart rate (PHR), anaerobic threshold exercise load (Load AT), maximal workload (Load max), anaerobic threshold oxygen uptake (VO2 AT), peak oxygen uptake (VO2peak), maximum ventilation per minute (VE max), maximum oxygen pulse (VO2/HRmax), and maximum respiratory exchange rate. The formula for calculating the change rate of CPET indexes before and after intervention is: (post-intervention value-pre- intervention value)/pre-intervention value × 100%.

### Randomization and sample size calculation

**Figure 1:**
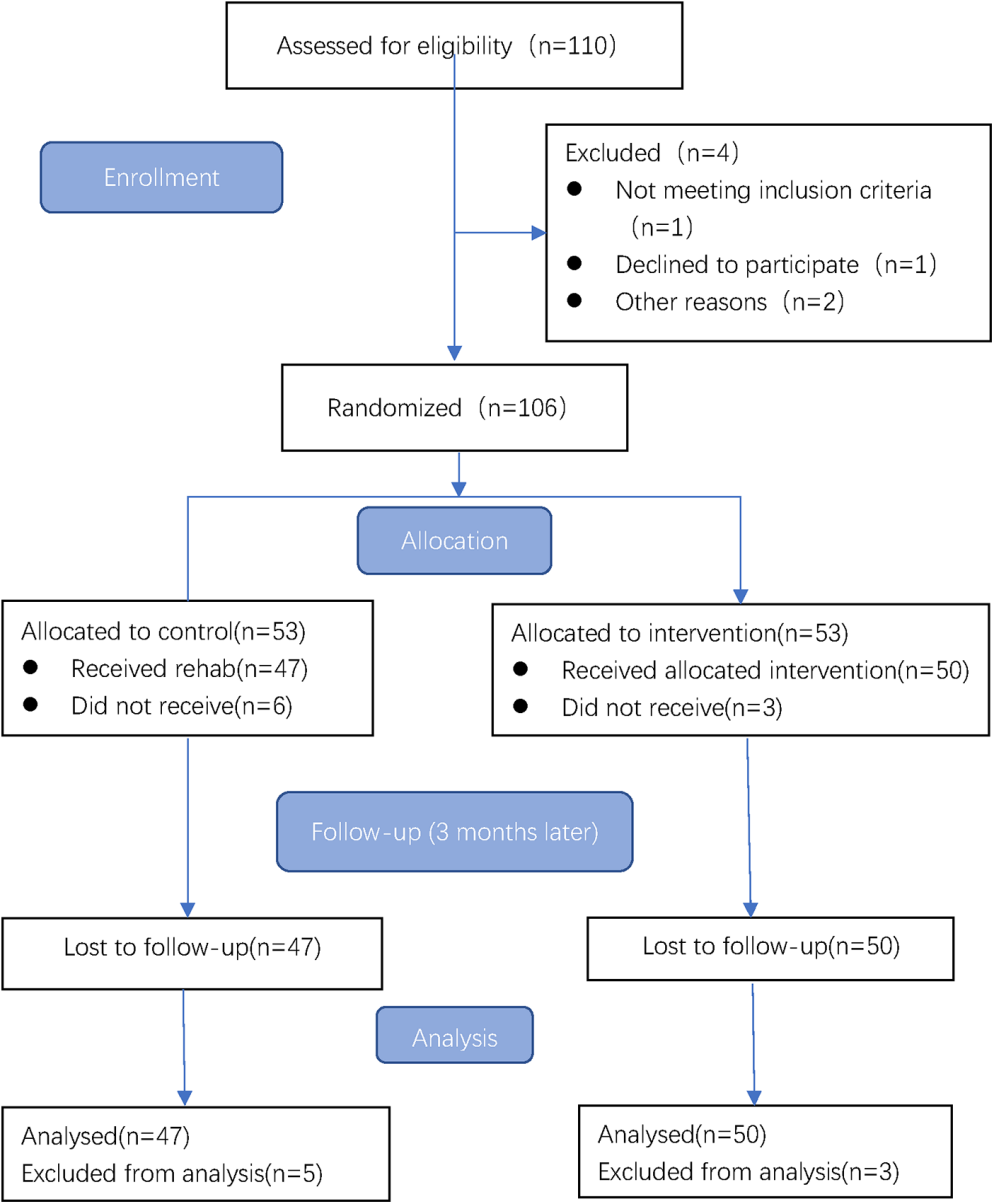
Study flow chart.

Random sequences were generated by researchers who were not involved in this study using the evenly distributed random number table in SPSS 20.0 and were divided into two groups. These values were then placed in sealed opaque envelopes numbered in sequence. The envelopes were given to a researcher not involved in data collection or patient assignment, and his only task was to group the patients randomly. Based on previous experiments (25), the calculated sample size was 42 participants per group (*n* = 84), with a significance level of 5%. To optimize the analysis of the results, we selected 53 participants per group, with a total sample size of 106 patients, considering an expected follow-up loss rate of 20%. The flowchart is shown in Figure 1.

### Statistical analysis

Statistical analysis was performed using SPSS version 20.0. The measurement data were tested for normality using the Shapiro–Wilk test, normally distributed data were expressed as mean ± standard deviation (x ± s) using the *t*-test, non-normally distributed data were expressed as percentile (lower quartile to upper quartile) using the Mann–Whitney *U*-test, and count data were expressed as percentages (%) using the *χ*2 test and Fisher's test. Differences were considered statistically significant when the *p*-value was <0.05.

## Results

In total, 106 patients were included in this study and randomized into groups A (home- based cardiac rehabilitation, *n* = 53) and B (center guided home-based rehabilitation, *n* = 53). There were 6 patients in group A and 3 patients in group B who were lost to follow-up. Finally, Group A had 47 patients and Group B had 50 patients, comprising 85 men and 12 women with a mean age of 57.06 ± 9.21 years. Table 1 summarizes the baseline characteristics of the two patient groups, and there were no significant differences between the two groups.

**Table 1:** Comparison of clinical data between the two groups.

Characteristic	A (*n *= 47)	B (*n *= 50)	*P*-value*
Age, year	58.85 (9.02)	55.38 (9.15)	0.063
Sex (% male)	40 (85.1%)	45 (90.0%)	0.464
Body mass index, kg/m^2^	25.64 (2.85)	26.36 (0.69)	0.101
Left ventricular ejection fraction, %	58.34 (4.30)	57.43 (3.27)	0.280
Low-density lipoprotein cholesterol, mmol/L	2.49 (0.77)	2.59 (0.70)	0.532
ACS type (*n*)			0.064
STEMI	41 (87.2%)	36 (72%)	
NSTE-ACS	6 (12.8%)	14 (28%)	
Hypertension (% yes)	24 (51.1%)	26 (52.0%)	0.927
Diabetes (% yes)	11 (23.4%)	21 (42.0%)	0.052
Hyperlipidemia (% yes)	32 (68.1%)	33 (66.0%)	0.827

ACS, acute coronary syndrome; STEMI, ST-segment elevation myocardial infarction; NSTE-ACS, non-ST segment elevation acute coronary syndrome.

Values are *n* (%) or mean (SD).

* Based on chi-square or t test as applicable.

There was no significant difference in CPET indices before and after the intervention in Group A (all *P* > 0.05), however, after the intervention in Group B, METs (max), PHR, Load AT, Load max, VE max, VO2peak, VO2 AT, and VO2/HR max were all higher than those before the intervention (all *P* < 0.05) (Table 2). In addition, before the intervention, we found that METs (max), Load AT, Load max, VO2 AT, and VO2peak showed no significant differences between the two groups (all *P* > 0.05); however, after the intervention, these indices were higher in Group B than in Group A (all *P* < 0.05) (Table 3). Table 4 showed that the change rates of VO2peak, METs(max), PHR, Load(max), VO2(AT), VE(max) and VO2/HR(max) in the two groups were significantly different before and after intervention (*P* <  0.05).

**Table 2:** Intra-group comparison of CPET indicators between the two groups before and after intervention.

Indicators	A (*n* = 47)		B (*n* = 50)		*P_1_*	*P_2_*
	Pre-intervention	Post-intervention	Pre-intervention	Post-intervention		
METs(max)	5.35 ± 0.90	5.35 ± 0.94	5.14 ± 1.12	6.36 ± 1.18	0.973	0.000
PHR, bpm	118.06 ± 14.50	117.91 ± 17.66	130.34 ± 18.39	134.48 ± 15.96	0.940	0.034
Load AT, w	69.96 ± 21.32	72.04 ± 20.05	76.96 ± 24.02	84.10 ± 21.65	0.451	0.000
Load max, w	121.13 ± 25.15	118.42 ± 24.96	111.46 ± 29.98	130.66 ± 34.80	0.281	0.000
VO2 AT, ml/min/kg	12.32 ± 2.80	12.67 ± 3.10	12.78 ± 3.49	14.74 ± 3.21	0.360	0.000
VO2peak, ml/min/kg	18.51 ± 3.39	18.74 ± 3.29	18.00 ± 3.91	22.26 ± 4.12	0.621	0.000
VE max, L/min	55.77 ± 12.88	54.38 ± 13.51	44.43 ± 10.88	54.28 ± 13.01	0.329	0.000
VO2/HR max, ml	11.84 ± 2.26	11.87 ± 2.22	10.43 ± 2.62	12.09 ± 2.89	0.901	0.000
RER max	1.26 ± 0.11	1.24 ± 0.13	1.15 ± 0.11	1.18 ± 0.07	0.445	0.103

CPET, Cardiopulmonary exercise test; METs, maximum metabolic equivalents; PHR, peak heart rate; Load AT, anaerobic threshold exercise load; Load max: maximal workload; VO2 AT: anaerobic threshold oxygen uptake; VO2peak: peak oxygen uptake; VE max: maximum ventilation per minute; VO2/HR max: maximum oxygen pulse.

Group A: home-based rehabilitation group; Group B: hospital-based rehabilitation group.

*P_1_*: Group A (pre-intervention) vs. Group A (post-intervention); *P_2_*: Group B (pre-intervention) vs. Group B (post-intervention).

**Table 3:** Comparison of CPET indicators between the two groups before and after intervention.

Indicators	A (*n* = 47)		B (*n* = 50)		*P_3_*	*P_4_*
	Pre-intervention	Post-intervention	Pre-intervention	Post-intervention		
METs(max)	5.35 ± 0.90	5.35 ± 0.94	5.14 ± 1.12	6.36 ± 1.18	0.330	0.000
PHR, bpm	118.06 ± 14.50	117.91 ± 17.66	130.34 ± 18.39	134.48 ± 15.96	0.000	0.000
Load AT, w	69.96 ± 21.32	72.04 ± 20.05	76.96 ± 24.02	84.10 ± 21.65	0.133	0.006
Load max, w	121.13 ± 25.15	118.42 ± 24.96	111.46 ± 29.98	130.66 ± 34.80	0.088	0.049
VO2 AT, ml/min/kg	12.32 ± 2.80	12.67 ± 3.10	12.78 ± 3.49	14.74 ± 3.21	0.392	0.002
VO2peak, ml/min/kg	18.51 ± 3.39	18.74 ± 3.29	18.00 ± 3.91	22.26 ± 4.12	0.493	0.000
VE max, L/min	55.77 ± 12.88	54.38 ± 13.51	44.43 ± 10.88	54.28 ± 13.01	0.000	0.977
VO2/HR max, ml	11.84 ± 2.26	11.87 ± 2.22	10.43 ± 2.62	12.09 ± 2.89	0.006	0.671
RER max	1.26 ± 0.11	1.24 ± 0.13	1.15 ± 0.11	1.18 ± 0.07	0.000	0.004

*P_3_*: Group A (pre-intervention) vs. Group B (pre-intervention); *P_4_*: Group A (post-intervention) vs. Group B (post-intervention).

**Table 4:** Comparison of the change rate of CPET indicators between the two groups before and after intervention.

Indicators	A (*n* = 47)	B (*n* = 50)	*P**-***value*
VO2peak, ml/min/kg	0.02(−0.07,0.10)	0.17 (0.11,0.39)	0.000
METs(max)	0.02(−0.10,0.10)	0.19 (0.11,0.40)	0.000
PHR, bpm	−0.02(−0.07,0.06)	0.04(−0.01,0.09)	0.010
Load AT, w	0.05(−0.15,0.18)	0.10 (0.05,0.22)	0.085
Load max, w	−0.0098 ± 0.0226	0.1871 ± 0.0205	0.000
VO2 AT, ml/min/kg	0.00(−0.11,0.17)	0.08 (0.03,0.39)	0.003
VE max, L/min	−0.01(−0.12,0.11)	0.22 (0.08,0.33)	0.000
VO2/HR max, ml	0.04(−0.11,0.10)	0.10 (0.03,0.26)	0.000
RER max	−0.01(−0.05,0.04)	0.02(−0.05,0.11)	0.151

The formula for calculating the change rate of CPET indexes before and after intervention is: (post-intervention value-pre-intervention value)/pre-intervention value  × 100%.

* Based on Mann–Whitney U or *t*-test as applicable.

## Discussion

The main results of this randomized controlled trial showed that cardiac exercise rehabilitation improved ventilation function, exercise endurance, and cardiopulmonary reserve capacity in patients with ACS who underwent complete coronary revascularization prior. Compared with the home-based cardiac rehabilitation model, METs (max), Load AT, Load max, VO2peak, and VO2 AT were higher in the center guided home-based rehabilitation group after the intervention, which seemed to indicate significantly improved cardiopulmonary function in a short period of time.

The CPET index, VE max, refers to the ventilation volume at maximum exercise intensity, which reflects the ventilation function of the patient. AT is a turning point when the exercise load increases to a certain degree; simple aerobic metabolism is no longer sufficient to meet the needs of the body, and anaerobic metabolism begins to contribute to energy supply. Exercise Load and METs (max) can effectively reflect exercise tolerance and intensity in patients. VO2 max refers to the maximum oxygen uptake capacity of an organism under limited exercise conditions. Together with VO2 AT, PHR, and VO2/HR max, VO2 max reflects cardiac reserve function. A decrease in this value indicates a decrease in cardiac reserve function and exercise tolerance (23). Our study showed that after the intervention, METs (max), Load AT, Load max, VO2peak, and VO2 AT values increased in the center guided home-based rehabilitation group compared with that before the intervention, indicating that regular exercise training improves the aerobic and anaerobic adaptability of the body, oxidation capacity of the skeletal muscle, and thus exercise tolerance (13). Simultaneously, it can enhance the contractility of the myocardium, thereby improving blood pumping ability, systolic and diastolic functions, and reserve ability of the heart.

Several studies (24–29) have demonstrated that among low-risk patients after myocardial infarction, revascularization, or heart failure, home- and hospital-based cardiac rehabilitation can achieve similar effects on exercise tolerance, quality of life, and clinical outcomes within 3–12 months. In a systematic review of the safety of home- based cardiac rehabilitation, Stefanakis et al. found that the incidence and severity of adverse events were very low (30). In a randomized controlled trial of home-based cardiac rehabilitation conducted by Raquel Bravo Escobar in patients with a moderate risk of ischemic heart disease, no serious cardiac complications were recorded, indicating the effectiveness of home-based cardiac rehabilitation, which can be used as an effective alternative model for hospital-based cardiac rehabilitation (17).

Unlike other home-based cardiac rehabilitation studies, we found no significant difference in CPET indices before and after the intervention in the home-based rehabilitation group. In this study, home-based exercise rehabilitation showed no significant short-term effect, which may be related to the difficulty of ensuring exercise quality during the intervention. Elderly individuals constituted a significant group in our study (*n* = 44). In the home-based rehabilitation process, China has low knowledge of home-based cardiac rehabilitation, which affects compliance and motivation for exercise rehabilitation. In addition to the lower acceptance and application ability of mobile technology, such as wearable devices, the lack of timely and standardized motor correction enables real-time evaluation and feedback of exercise skills and delays in solving problems, which fails to support themselves in standardized cardiac rehabilitation and affects the quality of exercise rehabilitation (31). The predominance of males in this study (*n* = 85) and poor lifestyle habits such as smoking and alcohol consumption affected the outcomes of cardiac rehabilitation. Remote supervision and management of home exercise rehabilitation through wearable devices and mobile medical technology support do not yet fully meet patient needs. Moreover, the intervention content is more limited, and dynamic changes in the disease stage and prescription adjustment of timely guidance are insufficient, which compromise the effectiveness of exercise rehabilitation.

The development of cardiac rehabilitation in China has been slow, and a survey of the current status of cardiac rehabilitation in nationwide hospitals showed that only 30(24%) of the 124 tertiary hospitals had cardiac rehabilitation services, with a low coverage rate and uneven development in the eastern and western regions. Factors affecting patient compliance include patient-specific factors, such as sex, age, education level, and health literacy and social factors such as small number of rehabilitation centers, unreasonable geographical distribution, inconvenient transportation, cost problems, insufficient social support, lack of a sound medical referral system, and medical personnel's recognition and knowledge of cardiac rehabilitation.

Based on the national conditions of China, family cardiac rehabilitation has great advantages, but there are also some limitations and problems to be solved. The lack of effective monitoring mechanisms and quality control systems to assess the accuracy of patients' use of relevant equipment, authenticity of the uploaded data, and process of rehabilitation make it difficult to ensure the quality of patients' home cardiac rehabilitation. In terms of social factors, there are no comprehensive laws or regulations related to home-based cardiac rehabilitation and no support from relevant medical insurance policies. The home-based model brings a greater challenge to the patience and sense of responsibility of the professional staff and the self-management ability of patients (32). Therefore, the development of home-based or remote cardiac rehabilitation models has been limited.

At present, in order to ensure the effectiveness and safety of cardiac rehabilitation and improve the participation rate and compliance of patients, we can take the hospital-led family cardiac rehabilitation model as the transition model, and gradually transition from hospital-oriented to family-oriented cardiac rehabilitation. In the future, we need to make full use of mobile medical care, develop intelligent mobile devices, applications, and platforms (33), improve the operability of software, and increase health education efforts to strengthen patients' knowledge of cardiac rehabilitation and improve patients' compliance and self-management. Most clinical trials on the effectiveness of home-based cardiac rehabilitation are small, single-center, short-term trials, and there are few studies with hospital-based cardiac rehabilitation as a control; therefore, more clinical evidence from large-scale, multi-center randomized controlled trials is needed to better guarantee the effectiveness of home-based cardiac rehabilitation (34).

This study has some limitations, such as the small number of cases, small sample size, and short follow-up period. Future studies with larger sample size and longer follow- up period are warranted. And there are more indicators for evaluating the efficacy of cardiac rehabilitation, and the differences in the efficacy of the two cardiac rehabilitation modalities on multiple aspects of patients with acute coronary syndromes still need more research.Another limitation was the lack of blinding among the research groups and investigators. However, according to the experimental design, the participants could not be deceived during the exercise test; therefore, a blind method could not be implemented.

In summary, exercise rehabilitation, as the core element of cardiac rehabilitation, can help improve patients' ventilatory function, cardiopulmonary endurance, and quality of life. In addition, the center guided home-based rehabilitation model seemed to show more remarkable improvement in cardiopulmonary function than the home-based cardiac rehabilitation model over a short period.

## Data Availability

The raw data supporting the conclusions of this article will be made available by the authors, without undue reservation.
